# Efficacy of Cilostazol on Uncontrolled Coronary Vasospastic Angina: A Pilot Study

**DOI:** 10.1111/j.1755-5922.2012.00312.x

**Published:** 2013-04-18

**Authors:** Sang-Yong Yoo, Sung-Gook Song, Jae-Hwan Lee, Eun-Seok Shin, Jeong-Su Kim, Yong-Hyun Park, Jun Kim, Kook-Jin Chun, June-Hong Kim

**Affiliations:** 1 Department of Internal Medicine, Division of Cardiology, University of Ulsan College of Medicine, Gangneung Asan HospitalGangneun, South Korea; 2 Department of Cardiology, HanMaeum Hopspital Changwon CityChangwon, South Korea; 3 Cardiovascular Center, Chungnam National University HospitalDaejeon, South Korea; 4 Division of Cardiology, Ulsan University Hospital, University of Ulsan College of MedicineUlsan, South Korea; 5 Cardiovascular Center, Pusan National University Yangsan HospitalYangsan, South Korea

**Keywords:** Cilostazol, Coronary vasospasm, Treatment

## Abstract

**Background:**

Although an angina attack by vasospastic angina (VSA) can usually be relieved or controlled with nitrates and calcium channel blockers (CCBs), there are some patients who cannot be controlled even by higher doses and combinations of these drugs. Cilostazol is a selective inhibitor of phosphodiesterase 3 that increases intracellular cyclic adenosine monophosphate (cAMP) contents. A stimulation of cAMP signal transduction increases coronary nitric oxide production. We examined whether cilostazol improved angina symptoms in patients with VSA uncontrolled by conventional treatment.

**Methods:**

This study was conducted in a prospective, multicenter, nonrandomized manner. The subject consisted of 21 patients (13 men, 57 ± 9 year-old) who were diagnosed with VSA and had at least two angina attacks during the past 1 week despite of conventional medications such as CCBs and/or nitrates. They took cilostazol 100 mg twice daily for 2 weeks in addition to the conventional medications. The patients recorded the frequency of angina attack and wrote down the numeric rating scale of a "severity of angina attack" while taking conventional medications and cilostazol for 2 weeks, and also recorded an averaged scale or total number of event during the last week at the time of the assessment. Using the Wilcoxon rank-sum test, we compared the changes in the scores of frequency and severity of angina attack before and after adding cilostazol to the conventional medications.

**Results:**

After adding cilostazol to the conventional medications, there were 78.9% relative reduction of the score of angina intensity and 73.5% of angina frequency (*P* < 0.001). There were four patients (19%) who were forced to stop cilostazol due to headache as an adverse event.

**Conclusions:**

Cilostazol appears to be an effective therapy in VSA uncontrolled with conventional medical treatment. A further prospective, randomized, placebo-controlled study will be needed to validate this result.

## Introduction

Although an angina attack by vasospastic angina (VSA) can usually be relieved or controlled with nitrates and calcium channel blockers (CCBs), there are some patients who cannot be controlled even by higher doses and combinations of these drugs. In addition, adverse effects such as headache, hypotension, or pedal edema limit the use of these drugs. These uncontrolled VSA patients may have a risk of lethal arrhythmias or sudden cardiac death. Even through the use of an implantable cardioverter defibrillator for ventricular tachycardia and ventricular fibrillation during coronary spasm attacks in intractable VSA may be considered as a nondrug treatment, no consensus has been established concerning the validity of this treatment in patients who can be prevented with drug therapy [1]. If it is not possible to control of coronary spasm with CCBs or nitrates, a drug that has a different mechanism of action is required.

Intracellular cyclic adenosine monophosphate (cAMP) signal transduction plays an important role in the regulation of endothelial nitric oxide (NO) production, and stimulation of cAMP signal transduction increases coronary NO production [2]. Cilostazol (Otsuka Pharmaceutical Co. Ltd., Tokushima, Japan), 6-[4-(1-cyclohexyl-1H-tetrazol-5-yl) butoxy]-3, 4-dihydro-2 (1H)-quinolinone, is a selective inhibitor of phosphodiesterase 3 (PDE3) that increases intracellular cAMP contents. Previous studies have suggested that cilostazol has various pleiotropic effects, such as NO dependent vasodilation, antiplatelet action, and improvements of endothelial dysfunction as well as antiinflammatory, antiproliferative, and antioxidative effects [3,4]. Therefore, we examined whether cilostazol improves angina symptoms in patients with VSA uncontrolled by conventional treatment.

## Materials and Methods

### Study Subjects

This trial was conducted according to the principles of the Declaration of Helsinki regarding investigation in humans, and was approved by the Institutional Review Board of each participating hospital. This study was conducted in a prospective, multicenter cohort manner. The subject consisted of 21 patients (13 men) who were diagnosed with VSA and had at least two angina attacks during the past 1 week in spite of conventional medications such as CCBs and/or nitrates. The diagnosis of VSA was made when at least one of the following criteria was met: (1) resting angina associated with ST-segment elevation of at least 2 mm in at least two electrocardiogram (ECG) leads; (2) epicardial coronary spasm documented by coronary angiography during spontaneous angina or provoked by intracoronary or intravenous ergonovine; and (3) positive result of ergonovine provoked echocardiography (Table 1). Exclusion criteria were the followings; (1) if there is an evidence of significant de-novo coronary artery or in-stent restenosis defined as greater than 50% diameter stenosis on coronary angiogram, (2) if there is an evidence of a positive stress test such as an exercise ECG test or a myocardial scintigraphic perfusion scan. Informed consent was obtained from each patient. The study protocol was approved by the Ethics Committee.

**Table 1:** Confirmatory diagnostic tests for vasospastic angina

	N = 21
Spontaneous spasm	9 (42.9%)
Confirmed by ECG	3 (14.3%)
Confirmed by CAG	6 (29.0%)
Ergonovine provoked CAG	9 (42.9%)
Ergonovine provoked ECHO	3 (14.3%)

CAG, coronary angiography; ECG, electrocardiography; ECHO, echocardiography.

### Diagnostic Coronary Angiography and Ergonovine Provocation Test

**Figure 1:**
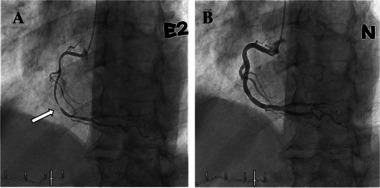
Representative example of ergonovine provocation testing during diagnostic coronary angiography in an 72-year-old man with vasospastic angina. Intravenous injection of ergonovine (E2) provoked subtotal occlusion of the mid portion of the right coronary artery (arrow) (**A**), and the angiogram after injection of intracoronary nitrate showed near normal right coronary artery and relief of total occlusion (**B**).

Except for sublingual nitroglycerin, all antianginal mediations including CCBs, nitrates, and beta-blockers were discontinued at least 3 days before coronary angiography. Except two cases that underwent both "exercise ECG test" and "ergonovine echocardiogram," diagnostic coronary angiography was performed in 19 patients (90.4%) by the Judkins technique. After coronary angiography was performed, in the cases that did not have other objective evidence of spasm, graded doses (50, 100, and 200 μg for intravenous provocation, or 1, 5, 10, and 30 ug diluted in volumes of 5 to 10 mL of normal saline for intracoronary provocation) of ergonovine were injected in succession at least at 3-min intervals until coronary spasm was induced or maximal dose was attained. Coronary angiograms were obtained after each dose. Finally, angiograms were obtained in several projections after the injection of 2–5 mg of intracoronary nitrate into both the left and right coronary arteries, and the organic coronary artery lesion was evaluated. A 12-lead ECG and arterial blood pressure were continuously monitored during the study. A 12-lead ECG was recorded at each stage. For the prevention of delayed spasm, all subjects were given 10 mg of short-acting nifedipine sublingually after finishing the test. A temporary pacemaker was not routinely used in all patients during the provocation test. A positive spasm was defined as transient >75% narrowing of a segment of coronary artery with symptoms or signs of myocardial ischemia (e.g., ST-segment depression, ST-segment elevation), and then the narrowed segments were recovered either spontaneously or after induction by intracoronary nitrate with restoration of the symptoms or signs to the resting condition (Figure 1).

### Ergonovine Provoked Echocardiography

An intravenous line was placed in the upper arm, and noninvasive blood pressure and lead II of the ECG were monitored during the entire procedure. Bolus injections of graded doses of ergonovine (150 and 200 μg) were administered intravenously at 3-min intervals until a positive response was obtained or a total dose of 350 μg was reached. The 12-lead ECG was recorded after each ergonovine injection and left ventricular wall motion was monitored continuously. Positive criteria for the test included the appearance of transient ST-segment elevation or depression at least 2 mm in at least two ECG leads or reversible regional wall motion abnormalities by 2D echocardiography. An intravenous bolus injection of nitroglycerin (200 μg) and sublingual nitroglycerin (0.6 mg) were given as soon as a positive response was detected or a negative response at the end of the test [5].

### Study Protocol

Once the patients who were diagnosed with VSA had at least two angina attacks during the past 1 week despite conventional medications such as CCBs and/or nitrates, they took cilostazol 100 mg twice daily for 2 weeks as in addition to the conventional medications. A questionnaire for detailing frequency and severity of angina attack was given to the patient at the initiation of the study. The patients recorded the frequency of angina attack and wrote down the numeric rating scale of a "severity of angina attack" in the questionnaire while taking conventional medications and cilostazol for 2 weeks, and also recorded an adverse event of cilostazol in the questionnaire. Each "chest pain scale" or "frequency" was expressed as an average or total number of event during the last week at the time of the assessment. The numeric rating scale of the "severity of angina attack" was a 0–10 scale, where the scale 0 was defined as no pain at all, while the scale 10 was defined as the worst pain imaginable (Wong Baker Faces Pain Scale) [6].

### Statistical Analysis

Continuous variables are expressed as mean ± standard deviation and categorical variables as number and percentages. Frequency and severity of angina attack were analyzed by the Wilcoxon rank-sum test using the final baseline difference in median value. A value of *P* < 0.05 was considered statistically significant. All the statistical analyses were performed with the Statistical Package for Social Science software 12.0 (SPSS 12.0 for Windows, SPSS Inc. Chicago, IL, USA).

## Results

As a method of diagnostic test for VSA, nine patients with spontaneous spasm were confirmed by electrocardiogram or coronary angiography. Ergonovine provoked coronary angiography or echocardiography was used for the confirmation of the rest of the patients (Table 1). The baseline clinical characteristics of 21 patients are shown in Table 2. Three patients have received coronary angioplasty with stent implantation due to significant coronary artery disease.19 (90.5%) patients have been taking diltiazem, and about half of the patients have been taking long-acting nitrates. Among patients, 19 (90.5%) patients underwent coronary angiography, and none of them had significant de novo coronary artery disease (≥50% diameter stenosis) or in-stent restenosis. The spasm occurred most often in the left coronary artery or left anterior descending artery territory (12 cases, 57.1%), and two patients (9.5%) had spasm in multivessels territories (Table 3).

**Table 2:** Baseline clinical characteristics of all study patients

Variables	N = 21
Male (gender)	13 (61.9%)
Age (years)	57.0 ± 8.7
Height (cm)	163.7 ± 9.1
Weight (kg)	64.9 ± 8.8
BMI	24.2 ± 2.6
Systolic blood pressure (mmHg)	125.7 ± 16.0
Diastolic blood pressure (mmHg)	80.6 ± 11.1
Pathologic Q wave	0 (0%)
Hypertension	8 (36.4%)
Diabetes mellitus	3 (13.6%)
Current/ex-smoking	9 (40.9%)
Previous CAD	4 (18.2%)
Previous MI	1 (4.5%)
Previous stroke	1 (4.5%)
Previous PCI	3 (13.6%)
Previous CABG	1 (4.5%)
Previous heart failure	0 (0%)
Chronic renal failure	0 (0%)
Chronic lung disease	0 (0%)
Creatinine (mg/dL)	1.0 ± 0.1
Total cholesterol (mg/dL)	155.0 ± 38.8
TG(mg/dL)	86.9 ± 31.9
HDL-C(mg/dL)	53.9 ± 12.8
LDL-C(mg/dL)	96.8 ± 33.0
hs-CRP (mg/dL)	1.1 ± 2.4
EF (%)	63.1 ± 5.5
Medications	
Nitrate	11 (52.4%)
DHP-CCB	8 (38.1%)
Diltiazem	19 (90.5%)
Nicorandil	6 (28.6%)
Statin	10 (47.6%)
Aspirin	8 (38.1%)
Beta-blocker	1 (4.8%)

BMI, body mass index; CABG, coronary artery bypass graft; CAD, coronary artery disease; HDL-C, high-density lipoprotein cholesterol; hs-CRP; high-sensitive C-reactive protein; LDL-C, low-density lipoprotein cholesterol; MI, myocardial infarction; PCI, percutaneous coronary intervention; TG, triglyceride.

**Table 3:** Severities of fixed coronary artery disease and spasm locations

	n (%)
Severities of fixed stenosis (data available in 19 patients)	n = 19
Normal	5 (26.3)
Luminal irregularities	4 (21.0)
Minimal stenosis (DS < 40%)	8 (42.1)
Intermediate stenosis (40%≤ DS < 75%)*	2 (11.0)
Significant stenosis (DS ≥ 75%)	0 (0)
Spasm documented	n = 21
In LAD (or LAD territory)	12 (57.1)
In LCX	5 (23.8)
In RCA (or RCA territory)	5 (23.8)
In RI	1 (4.8)
In multivessels spasm	2 (9.5)

DS, diameter stenosis; LAD, left anterior descending artery; LCX, left circumflex artery; RCA, right coronary artery; RI, ramus intermedius.

* Each case had 43%, 46% of diameter stenosis, respectively.

**Figure 2:**
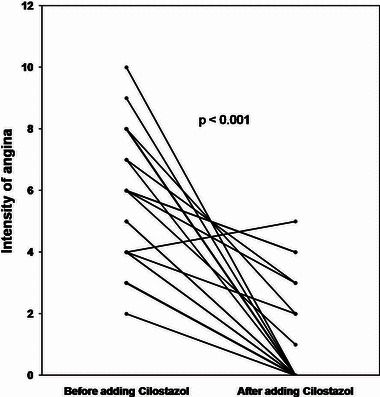
Changes of intensity of angina attack before and after adding cilostazol (n = 21). Mean intensity score of angina attack before and after adding cilostazol were 5.6 ± 2.2 and 1.1 ± 1.7, respectively. Wilcoxon signed ranks test resulted in *P* < 0.001.

**Figure 3:**
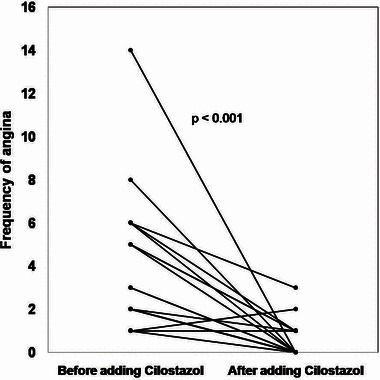
Changes of frequency of angina attack before and after adding cilostazol (n = 21). Mean frequency scoresof angina attack before and after adding cilostazol were 3.8 ± 3.1 and 0.5 ± 0.8, respectively. Wilcoxon signed ranks test resulted in *P* < 0.001.

**Figure 4:**
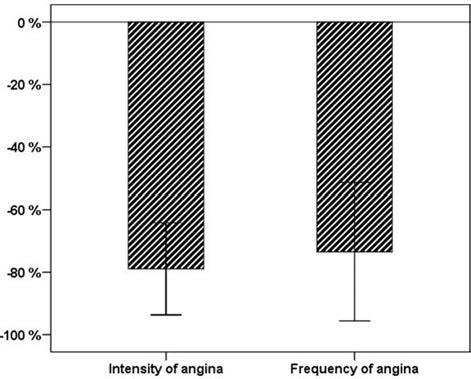
Relative reductions in the scores of intensity and frequency of angina attack after adding cilostazol to the conventional treatments (n = 21). The bar chart displayed astandard error representing a 78.9% relative reduction of the score of angina intensity and a 73.5% of the score of angina frequency.

The intensity of soreness due to angina attack decreased significantly (*P* < 0.001) for 2 weeks of cilostazol therapy (Figure 2), and the frequency of angina attack also decreased significantly (*P* < 0.001) (Figure 3). There were 78.9% and 73.5% of relative reduction in scores of the intensity and frequency of angina attack after adding cilostazol to the conventional therapy, respectively (Figure 4). The brief summary for all study patients is given in Table 4. All but one (No. 20) improved in the intensity of angina attack. Thirteen (70.0%) patients did not have an angina attack at all after adding cilostazol to the baseline medications. The frequency of angina attack decreased in 19 (90.5%) patients. Although, there were no changes in the frequency of angina attack in two (No. 9, 17) patients and an increase in one patient (No. 19), most of the patients had a decreased frequency of angina attack. However, no one has worsened in both intensity and frequency of angina attack in synchrony.

**Table 4:** Summary of study patients

No.	Age	Gender	Type of VAP	Diagnostic tool	Provocation tools	Sites of spasm	Medications before adding cilostazol	Intensity of angina attack	Frequency of angina attack
1	51	F	Pure	ergECHO	Ergonovine	LAD	Diltiazembid/nitrate bid	5→ 0	2→ 0
2	55	M	Pure	CAG	Spontaneous spasm	LCX	Diltiazem bid/nitrate bid	5→ 0	2→ 0
3	71	M	Mixed	ergCAG	Ergonovine	LCX	Diltiazem bid	2→ 0	6→ 0
4	55	M	Pure	ergCAG	Ergonovine	LCX, RCA	DHP-CCB qd/diltiazemqd /nitrate qd	3→ 0	2→ 0
5	70	M	Pure	ergCAG	Ergonovine	RCA	DHP-CCB bid/diltiazem bid/ nitrate bid	8→ 0	2→ 0
6	49	F	Pure	ergECHO	Ergonovine	LAD	DHP-CCB qd/diltiazem bid/nitrate bid	8→ 0	14→ 0
7	64	M	Pure	ergCAG	Ergonovine	LCX, RI	DHP-CCB qd	3→ 0	3→ 0
8	50	M	Pure	CAG	Spontaneous spasm	LAD	Diltiazem bid/nicorandil bid	3→ 0	1→ 0
9	72	M	Pure	ECG	Spontaneous spasm	LAD	Diltiazem bid/nitrate bid/nicorandil bid	4→ 2	1→ 1
10	61	M	Pure	CAG	Spontaneous spasm	RCA	DHP-CCB qd	7→ 0	2→ 0
11	47	F	Pure	ergECHO	Ergonovine	LAD	Diltiazem bid/nitrate qd/nicorandil bid	4→ 0	5→ 0
12	71	M	Mixed	ergCAG	Ergonovine	LAD	Diltiazem bid/nitrate bid/nicorandil bid/DHP-CCB qd	7→ 3	5→ 1
13	54	F	Pure	CAG	Spontaneous spasm	RCA	Diltiazem bid/nitrate qd/nicorandil bid	4→ 0	2→ 0
14	49	F	Mixed	CAG	Spontaneous spasm	LAD	Nicorandil bid/DHP-CCB qd	10→ 0	8→ 0
15	44	F	Pure	ECG	Spontaneous spasm	LAD	DHP-CCB qd/diltiazem bid/nitrate qd	6→ 3	6→ 3
16	50	M	Pure	CAG	Spontaneous spasm	LAD	Diltizem bid	6→ 4	6→ 1
17	51	F	Pure	CAG	Spontaneous spasm	RCA	Diltiazem bid	6→ 1	1→ 1
18	55	M	Mixed	ergCAG	Ergonovine	LAD	Diltiazem bid	9→ 0	3→ 0
19	56	F	Mixed	ergCAG	Ergonovine	LAD	Diltiazem bid	6→ 4	1→ 2
20	55	M	Pure	ergCAG	Ergonovine	LAD	Diltiazem bid	4→ 5	2→ 1
21	66	M	Pure	ergCAG	Ergonovine	LCX	Diltiazemqd/nitrate qd/nicorandilqd	8→ 2	5→ 1

Bid, twice a day; CAG, coronary angiography; ECG, electrocardiography; DHP-CCB, dihydropyridine calcium channel blocker; ergCAG, ergonovine provoked coronary angiography; ergECHO, ergonovine provoked echocardiography; F, female; M, male; qd, once a day.

Adverse reactions (side effects) are presented in Table 5. The most common adverse event was headache, followed by palpitation and/or tachycardia. Despite being angina-free after adding cilostazol, 4 of 5 (19.0%) patients who had headache were forced to stop cilostazol for 2 weeks during the study period. No patient was withdrawn during the course of protocol because of palpitation, tachycardia, or other reasons.

**Table 5:** Adverse events in 21 study patients

	N = 21
Headache	5 (23.8%)
Palpitation/tachycardia	3 (14.3%)
Headache and palpitation/tachycardia	1 (4.8%)

## Discussion

CCB alone or in combination with long-acting nitrates is known to be an effective therapy for most patients with VSA. However, 5% to 30% of patients with VSA were refractory to full medical treatment with calcium antagonist and nitrates [7]. According to Ministry of Health, Labour and Welfare-commissioned study in Japan defines intractable VSA that cannot be relieved with two types of coronary vasodilators such as nitrates and CCBs. In the report, 921 of 2,251 patients with angina had VSA, and the rate of intractable VSA was 13.7% (126 of 2,251). In those cases, if CCBs or nitrates cannot control the coronary vasospasm, a drug that works in a different mechanism will be required.

Cilostazol is a quinolinone derivative and a selective PDE3 inhibitor. In humans, the PDE3 isoenzyme is mainly found in platelets, vascular smooth muscle, liver, smooth muscle of the airway, T lymphocytes, adipose tissue, and cardiac tissue [8]. The inhibition of PDE3 leads to suppress cAMP degradation with a consequent increase in cAMP in platelets and blood vessels, resulting in inhibition of platelet aggregation and vasodilation, respectively [9,10]. In addition, cilostazol has pleotropic effects, including the improvement of serum lipid profile with lowering triglycerides, the elevation of high-density lipoprotein cholesterol, and the inhibition of vascular smooth muscle cell growth. Therefore, these favorable effects of cilostazol have been translated into preventing neointimal formation following angioplasty [11]. As the mechanism of vasodilating action by cilostazol has been demonstrated, several studies reported its effect of increasing nitric oxide production via a cAMP/PKA- and PI3K/Akt-dependent mechanism in a dose-dependent manner [12,13]. Cilostazol has therefore been used as a vasodilator in addition to antiplatelet drug for improving the ischemic symptoms in chronic peripheral arterial disease and preventing recurrence of cerebral infarction [14,15].

Although the exact mechanism of VSA has not been elucidated, endothelial dysfunction is regarded as one of the most important underlying mechanisms. The current first-line treatment drugs for VSA, CCB, or nitrate, are not included in the repertoire of drugs to improve the "endothelial dysfunction" per se [16]. In contrast, an interesting study demonstrated that cilostazol actually improved endothelial dysfunction by increasing coronary flow reserve and flow-dependent coronary dilation in patients with VSA [17].

Another important issue of VSA is whether or not to use aspirin. Controversy over aspirin in VSA still exists because of a dilemma between a subsequent thrombotic risk due to prolonged spasm and a possible chance of worsening spasm by prostacyclin blocking action of aspirin. Based on these considerations, it seems to be no reason not to use cilostazol for VSA because, theoretically, it is likely to have favorable effects for VSA not only in control of coronary vasospasm but also in prevention of thrombotic complication.

As a main result of our pilot study, adding cilostazol for patients with VSA who have not respond to conventional treatment including CCBs or nitrates causes to decrease the intensity of angina by 78.9% and the frequency of angina by 73.5% significantly. Because not all patients have fully received drugs-combination for coronary vasodilation, they could not be defined as an intractable VAS in the true sense of the word. Although our study was not a placebo-controlled trial or that comparing with other drugs, we reconfirmed the favorable effect of cilostazol on the intensity and frequency of angina attack in three (No. 7, 11, 16) patients. While they had angina attacks every day after stopping cilostazol, they completely disappeared after adding cilostazol in all three patients. We could not figure out which characteristics of the patients, among all patients with VSA, contributed to control coronary vasospasm after adding cilostazol.

## Conclusion

In this multicenter, prospective cohort study, cilostazol appears to be an effective therapy for VSA uncontrolled with conventional medical treatment, suggesting the potential applicability of cilostazol to VSA. A further large prospective, randomized, placebo-controlled study (STELLA) is now being conducted to validate this study results (http://www.clinicaltrial.gov/ct2/show/ NCT01444885?term=cilostazol+AND+vasospastic+ angina&rank = 1).
